# Replicative slender bloodstream forms complete transmission of *Trypanosoma brucei* without prior differentiation into stumpy forms

**DOI:** 10.7554/eLife.108688

**Published:** 2026-08-19

**Authors:** Carina Praisler, Jaime N Lisack, Anna Sophie Kreis, Laura Hauf, Johanna Odenwald, Fabian Imdahl, Markus Engstler

**Affiliations:** 1 Department of Cell and Developmental Biology, Biocenter, Julius-Maximilians-Universitaet Wuerzburg Germany; 2 Helmholtz Institute for RNA-based Infection Research, Helmholtz-Center for Infection Research Wuerzburg Germany; Centre for Molecular Biology of Heidelberg University (ZMBH) Germany; University of Geneva Switzerland

**Keywords:** kinetoplastida, trypanosomes, *Trypanosoma brucei*

## Abstract

We have previously shown that the slender form of *Trypanosoma (T.) brucei* is able to infect teneral tsetse flies, develop to the first fly form, which is the procyclic form, and complete the life cycle in the insect vector (Schuster et al., 2021). Further, analysis of the transmission index (TI; defined as the number of salivary gland infections relative to the number of midgut infections) revealed a higher TI for slender as compared to stumpy forms under laboratory conditions, which included the addition of *N*-acetylglucosamine (NAG) to the infective bloodmeal. Here, we show that slender trypanosomes can establish infections in both male and female tsetse flies and in both teneral and non-teneral flies without requiring supplements in the bloodmeal. Additionally, an RNA sequencing time course was performed on both slender and stumpy cells during their transition into procyclic forms. This analysis revealed that slender- and stumpy-form trypanosomes remain transcriptionally distinct throughout differentiation into the procyclic form. Furthermore, while the protein associated with differentiation 1 (PAD1) remains essential for the transition, slender cells do not require expression of other hallmark stumpy-form traits, such as cell-cycle arrest or the shortening of their flagella or microtubule corset. Instead, slender trypanosomes are able to transition directly into procyclic forms. Taken together, these findings demonstrate that slender cells of *T. brucei* can follow a distinct transcriptional trajectory towards the procyclic form and can establish infections in teneral and non-teneral tsetse flies, thereby contributing to the transmission and spread of these African parasites.

## Introduction

In the bloodstream of a mammalian host, two main forms of *Trypanosoma (T.) brucei* can be observed, the long slender and the short stumpy bloodstream form (bsf).

The slender cell is proliferative, whereas the stumpy cell has undergone cell-cycle arrest in G1/G0 phase. During division, slender cells release peptidases, most significantly oligopeptidase B and metallocarboxypeptidase 1 (Tettey et al., 2022), generating a pool of oligopeptides in the blood and tissues around them. These essential peptides, together with possible other unknown components, are part of the molecular cocktail collectively called the stumpy induction factor (SIF) (Vassella et al., 1997; Reuner et al., 1997; Bossard et al., 2013; Moss et al., 2015; Rojas et al., 2019). After exceeding a certain threshold, SIF causes the proliferative slender cells to change into the cell-cycle-arrested stumpy forms (Vassella et al., 1997; Reuner et al., 1997). This transition is accompanied by shortening of the flagellum, cell-cycle arrest, and other molecular changes, such as the remodelling of the mitochondrion to a cristate structure and expression of Krebs cycle enzymes. The trypanosomes also begin to express the protein associated with differentiation 1 (PAD1), a member of the carboxylate-transporter protein family, on their surface. PAD1 functions as a transducer of the signal that triggers differentiation into the procyclic form, the first fly form that develops in the tsetse midgut (Reuner et al., 1997; Dean et al., 2009).

Cell-cycle arrest is lethal for stumpy forms, as they die if they are not taken up by the tsetse fly within a few days. Thus, their purpose in the life cycle of *T. brucei* has long been debated, with the two most accepted theories being:

1. Stumpy forms are needed for the regulation of the parasitaemia in the host.
2. Due to pre-adaptation for life in the insect vector, they are the only form able to infect the tsetse fly (Robertson, 1912; Wijers and Willett, 1960; Fenn and Matthews, 2007; MacGregor et al., 2012; Matthews et al., 2015; Silvester et al., 2017).

In 2021, our laboratory reported unexpected plasticity in the life cycle of *T. brucei*. We found that even a single slender cell can infect the tsetse fly without exhibiting morphological and biochemical manifestations that define a stumpy cell (Schuster et al., 2021).

Instead, slender cells turn on the essential PAD1 pathway (Dean et al., 2009) and transit directly into procyclic forms – all whilst continuously dividing. We suggested that the ability of slender bloodstream forms to infect the tsetse fly vector would, at least in part, solve the transmission paradox, which refers to the low blood parasitaemia observed in chronically infected hosts and the small bloodmeal volume of tsetse flies, both of which make it unlikely that a stumpy form would be ingested (Capewell et al., 2019).

However, our original study was criticised (Matthews and Larcombe, 2022; Ngoune et al., 2025) for mainly four reasons:

1. As routinely done in tsetse laboratories, we supplemented all infectious bloodmeals with the immune-suppressive chemical *N*-acetylglucosamine (NAG), which is known to enhance tsetse midgut infections for stumpy forms but has no effect on subsequent salivary gland infections (Peacock et al., 2012). It was argued that this treatment allowed slender forms to infect the fly.
2. We used teneral flies (which have not yet taken a bloodmeal and are younger than 3 days) for all infection experiments. This led to the question if the slender trypanosomes might just be able to infect young flies.
3. The use of male flies is common practice for studying trypanosome infections. Nevertheless, could it be possible that slender trypanosomes can infect male but not female tsetse flies?
4. We had shown that slender trypanosomes express the differentiation marker *pad1* while becoming procyclic. This was taken as proof that slender cells must turn into stumpy cells before becoming procyclic.

To address the critique further than already done (Lisack et al., 2022), we have systematically conducted additional experiments. We found that slender-form trypanosomes can indeed infect both teneral and non-teneral flies (i.e. flies that have taken at least one non-infectious bloodmeal and are more than 72 hr post eclosion [hpe]), even in the absence of immune-suppressing chemicals.

Using an RNA sequencing time course, we confirmed that slender forms are able to transition directly to procyclic forms without becoming stumpy forms.

Taken together, these new findings further highlight the plasticity in the life cycle of *T. brucei*, as well as the ability of slender trypanosomes to contribute to the spread of these parasites to new hosts.

## Results

It has been shown that NAG aids stumpy trypanosome infection of the fly midgut, while not influencing the number of subsequent salivary gland infections (Peacock et al., 2006; Peacock et al., 2012).

To exclude the possibility that the use of the lectin-inhibitor NAG rendered the tsetse fly midgut artificially permissive to slender trypanosome infections, teneral tsetse flies were infected with slender cells in either untreated blood or NAG-supplemented blood.

As described in Schuster et al., a stumpy marker cell line (NLS-GFP:PAD1 3′UTR) was used to confirm that less than 1% PAD1-positive cells were present in any slender culture.

**Figure 1.**
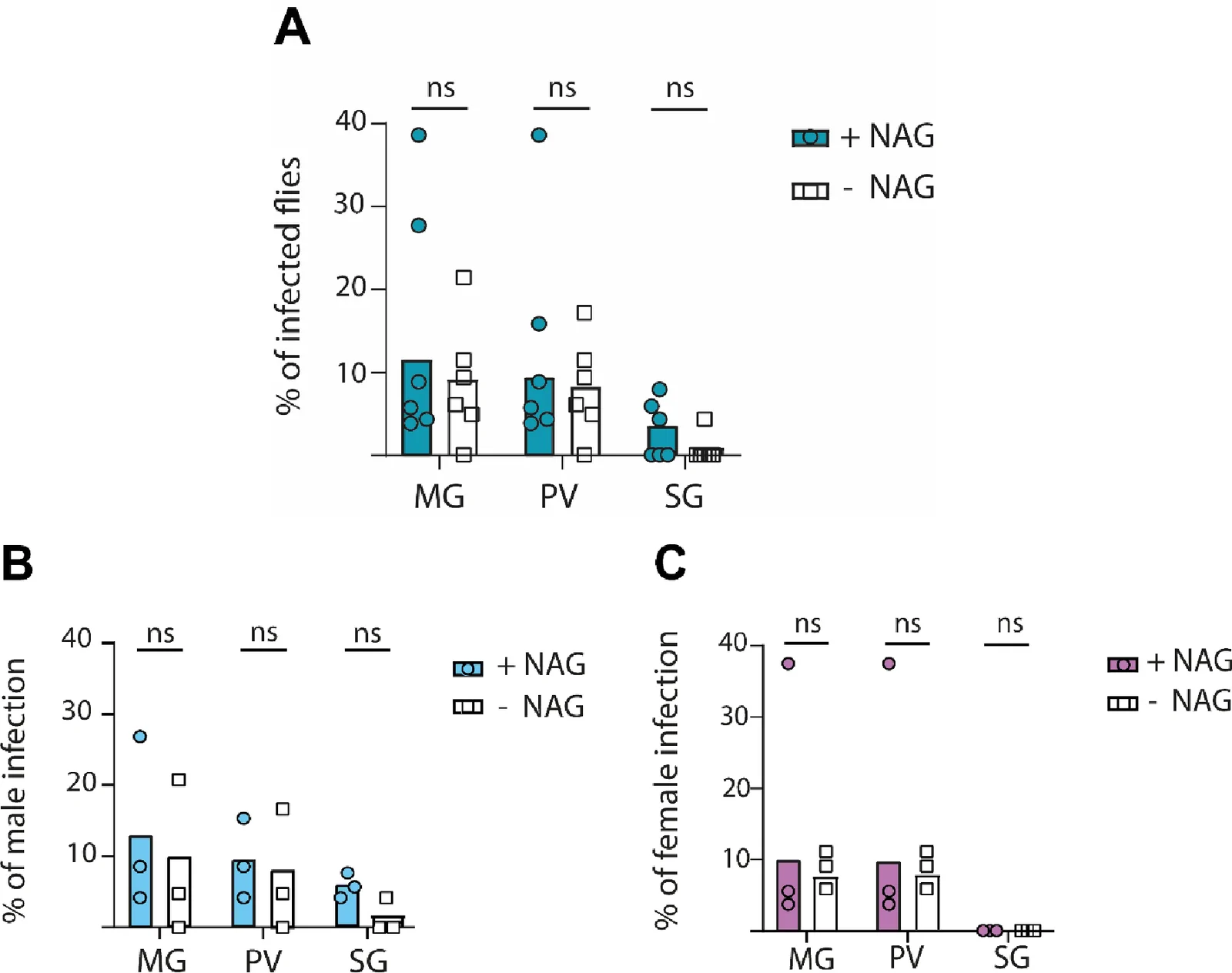
Infection rates (%) of slender *T.* *brucei* in teneral tsetse flies, with and without *N*-acetylglucosamine (NAG) supplementation. Flies of both genders were fed 200 slender cells per ml, translating to 4 slender cells per bloodmeal, estimating a 20 µl drinking volume for each tsetse fly (Gibson and Bailey, 2003), with or without 60 mM NAG. Slender cells were harvested and checked for PAD1 signal to confirm slender identity before infection (<1% PAD1 positive). Infections were performed in triplicates with approximately 20 flies/replicate. The midgut (MG), proventriculus (PV), and salivary glands (SG) of all flies were dissected after 35 days to check for infection. Bar graphs show mean infection rates across replicates, with individual dots representing infection percentages per replicate. Fisher’s exact test was used on the mean infection rates to determine significance. ns = not significant (p>0.05). Infection rates are shown for both sexes (**A**), male (**B**), and female flies (**C**).

Infection rates in the midgut and proventriculus were similar between non-supplemented and NAG-supplemented infections (Figure 1A and Supplementary file 1A). Midgut infections reached 11.6% with and 9.2% without NAG (Figure 1A) while proventriculus infections occurred in 9.4% and 8.3%, respectively (Figure 1A). Salivary gland infections were observed in 3.6% of flies infected with and 0.9% without NAG; however, this difference was not statistically significant (p>0.05) (Figure 1A).

The use of male flies is common practice for studying tsetse infections, as they show higher salivary gland infection rates than female flies (Jackson, 1949; Maudlin et al., 1990; Maudlin, 1991). However, because female flies have a longer lifespan, they cannot be disregarded as potential important contributors to parasite transmission in the wild (Maudlin et al., 1990). Therefore, the influence of fly sex on infection rates was assessed by using the same dataset as in Figure 1 (Figure 1B and C). Using NAG-supplemented blood, 8.0% of male flies exhibited midgut, 5.8% proventriculus, and 3.6% salivary gland infections. In the absence of NAG, midgut, proventriculus, and salivary gland infections were observed in 5.5%, 4.6%, and 0.9% of male flies, respectively (Figure 1B). Fisher’s exact test revealed no significant differences in infection rates between NAG-supplemented and non-supplemented groups for male flies (Figure 1B).

Among female flies, 3.6% harboured midgut and 3.7% proventriculus infections, regardless of NAG supplementation (Figure 1C). No salivary gland infections were identified in female flies (Figure 1C). The total percentage of fly infections and their corresponding replicates are provided in Supplementary file 1A.

Collectively, these results show that slender parasites can infect tsetse flies and establish stable infections without reliance on immunosuppressive compounds.

Although infection rates for teneral flies are relatively low, commonly ranging between 10% and 30%, they are more susceptible to trypanosome infection than non-teneral flies (Peacock et al., 2012). Consequently, the use of teneral flies is standard practice in *T. brucei* infection experiments, and we have exclusively used them in our previous infections. It is possible, however, that in nature, older flies contribute more to the spread of trypanosomes than younger flies. As such, it was of interest to test if slender trypanosomes could also infect non-teneral flies (Hoof et al., 1937; Wijers, 1958; Otieno et al., 1983; Walshe et al., 2011; Matthews and Larcombe, 2022). Therefore, to ensure our flies would survive for at least 30 days to allow infection assessment, we defined our non-teneral flies as those infected between 144 and 168 hpe. These flies were fed two non-infectious bloodmeals prior to the third infectious bloodmeal, all spaced at least 2 days apart.

For these experiments, we again used the cell line containing the NLS-GFP:PAD1 3′UTR stumpy reporter and a cytoplasmic red fluorescent protein (td Tomato) (Reuter et al., 2023). To ensure pure populations (Schuster et al., 2021), fluorescence-activated cell sorting (FACS) was performed to separate PAD1-positive (stumpy) from PAD1-negative (slender) cells. To confirm sorting success, populations were subsequently examined by fluorescence microscopy for PAD1 signal. Stumpy cells were also sorted to remove any slender cells and to keep conditions constant.

Additionally, the growth of sorted slender cells was monitored in vitro to ensure that sorting did not affect parasite fitness by causing any stress (Quintana et al., 2021; Figure 2—figure supplements 1 and 2). Only then did we proceed with infections, using either sorted slender or sorted stumpy cells to infect male and female teneral flies. With the confidence of having pure slender or stumpy populations, we increased parasite concentrations to 1×10^6^ cells/ml for infection. Flies were dissected 30 days post infection and their organs examined for parasite presence.

**Figure 2.**
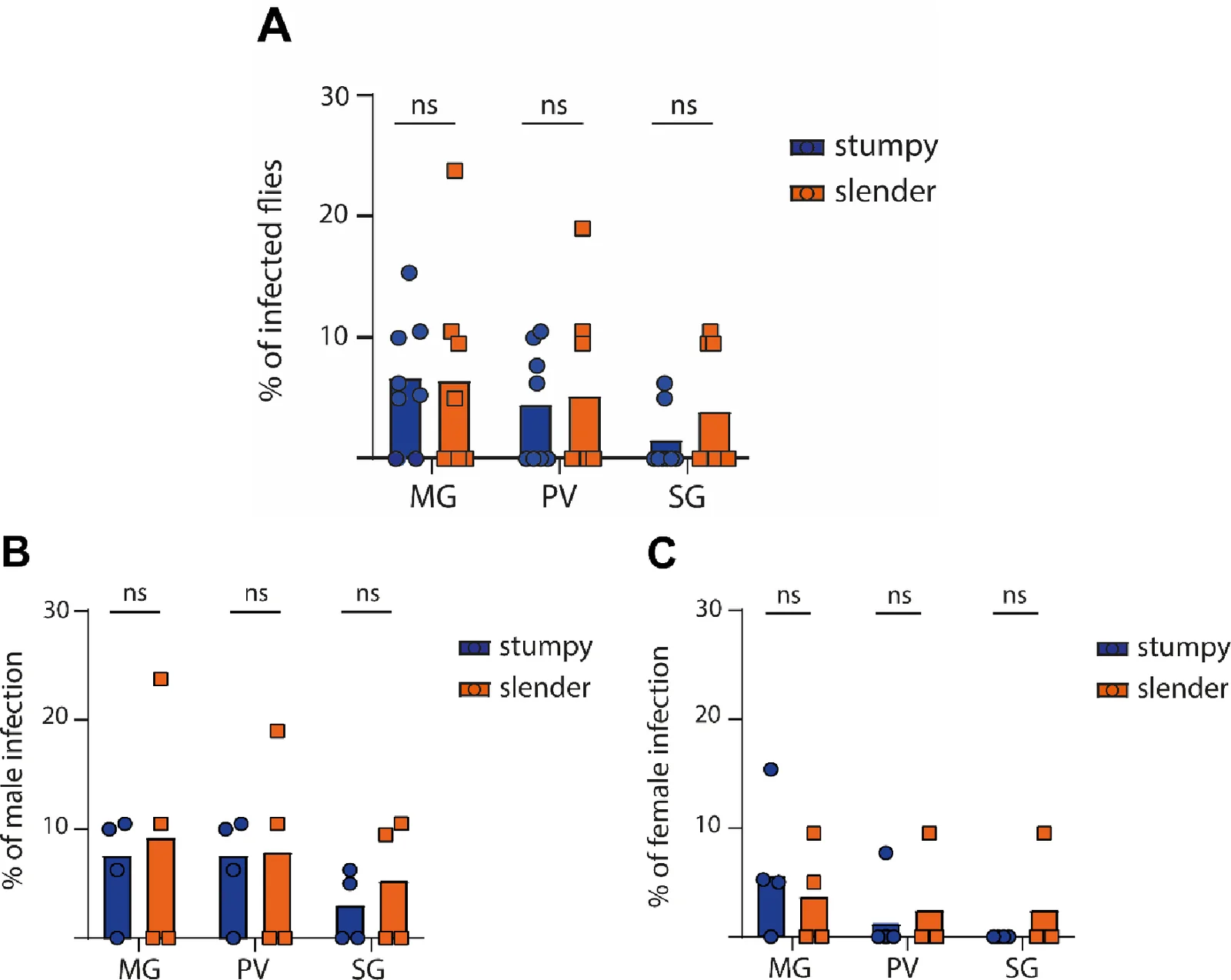
Infection rates (%) of slender and stumpy *T.* *brucei* in non-teneral tsetse flies. Non-teneral flies were infected between 144 and 168 hr post eclosion (hpe) with either slender (orange) or stumpy (blue) parasites, after receiving two non-infectious bloodmeals beforehand. The tdTomato NLS-GFP:PAD1 3′UTR cell line enabled fluorescence-activated cell sorting (FACS) to separate stumpy (PAD1 positive, GFP in nucleus) and slender (PAD1 negative, no nuclear fluorescence) prior to infection. With the confidence of pure slender or pure stumpy cultures after sorting, infection numbers were increased to 1×10^6^ cells/ml or 2×10^4^ cells per bloodmeal per fly, considering a 20 µl drinking volume per fly (Gibson and Bailey, 2003). Infections were performed in quadruplets with roughly 20 flies/replicate. Midgut (MG), proventriculus (PV), and salivary glands (SG) of all flies were dissected after 30–35 days to check for parasite presence. Bar graphs show mean infection rates across replicates, with individual dots representing infection percentages per replicate. Fisher’s exact test was used on the mean infection rates to determine significance; ns = not significant (p>0.05). Infection rates are presented for both sexes (**A**), males (**B**), and females (**C**).

Infection data show that slender trypanosomes were able to establish infections in non-teneral flies at rates comparable to stumpy forms (Figure 2A and Supplementary file 1B). Midgut infections were detected in 6.4% of flies infected with slender and 6.6% of flies infected with stumpy cells. Similarly, 5.1% and 4.4% of flies exhibited proventriculus infections, and 3.8% and 1.5% salivary gland infections, respectively. Statistical analysis using Fisher’s exact test revealed no significant difference for any fly organ in slender or stumpy infections.

**Figure 2—figure supplement 1.**
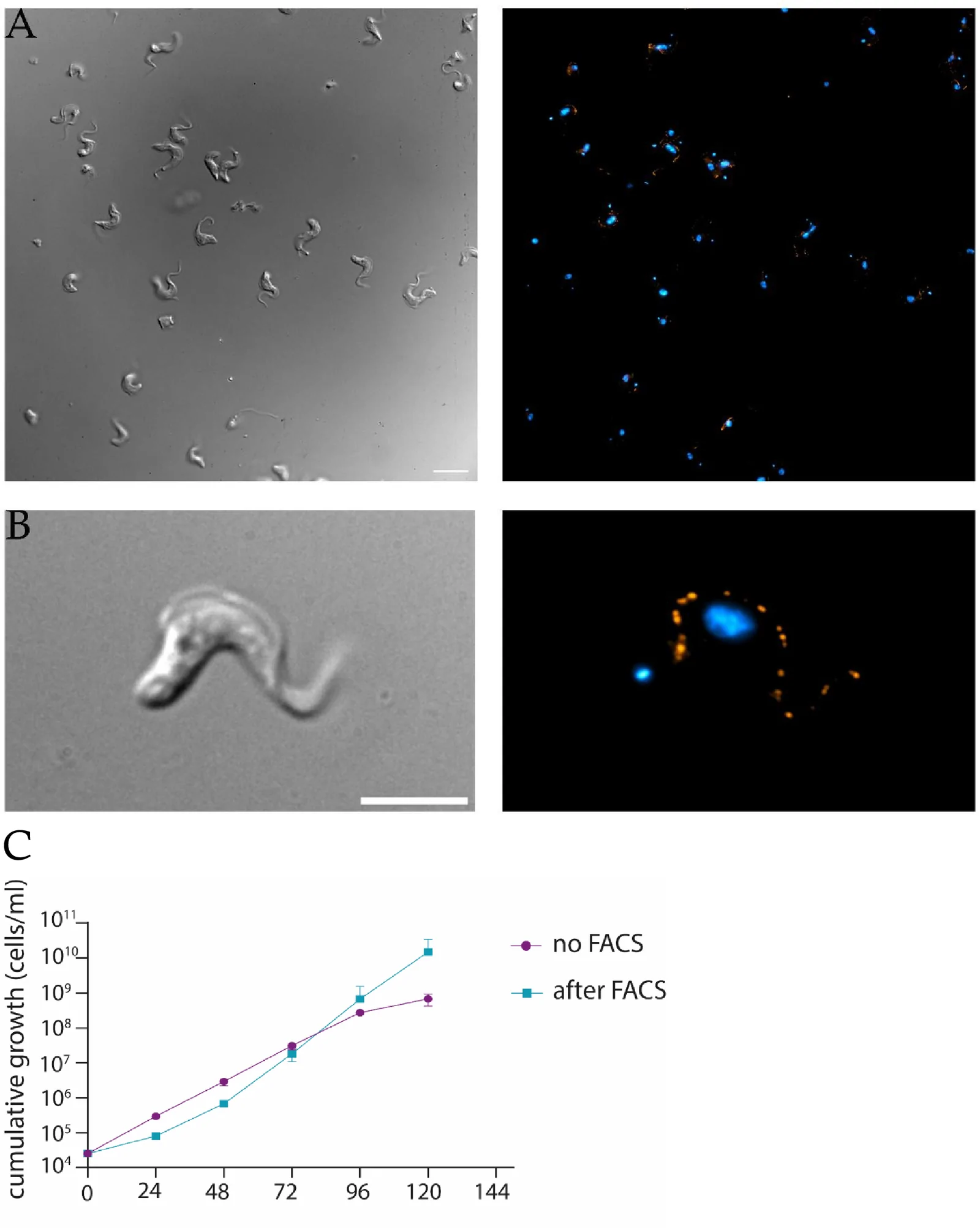
Slender *T. brucei* cells of the tdTomato NLS-GFP:PAD13′UTR line do not express PAD1 or exhibit stress responses following fluorescence-activated cell sorting (FACS). FACS was used to ensure pure slender populations (*pad1* negative) prior to infection. (**A**) Immunofluorescence (IF) images of slender cells immediately after FACS, confirming sorting success. Parasites were fixed in 4% paraformaldehyde (PFA), stained with DAPI (blue), and labelled with an anti-PAD1 antibody (orange); scalebar: 20 μm. (**B**) High-resolution IF image of a single slender trypanosome post sorting, showing clear absence of nuclear pad1 signal; scalebar: 10 μm. (**C**) Growth curves comparing slender cells post sorting (green) and untreated slender cells (purple), indicating no growth impairment due to sorting.

**Figure 2—figure supplement 2.**
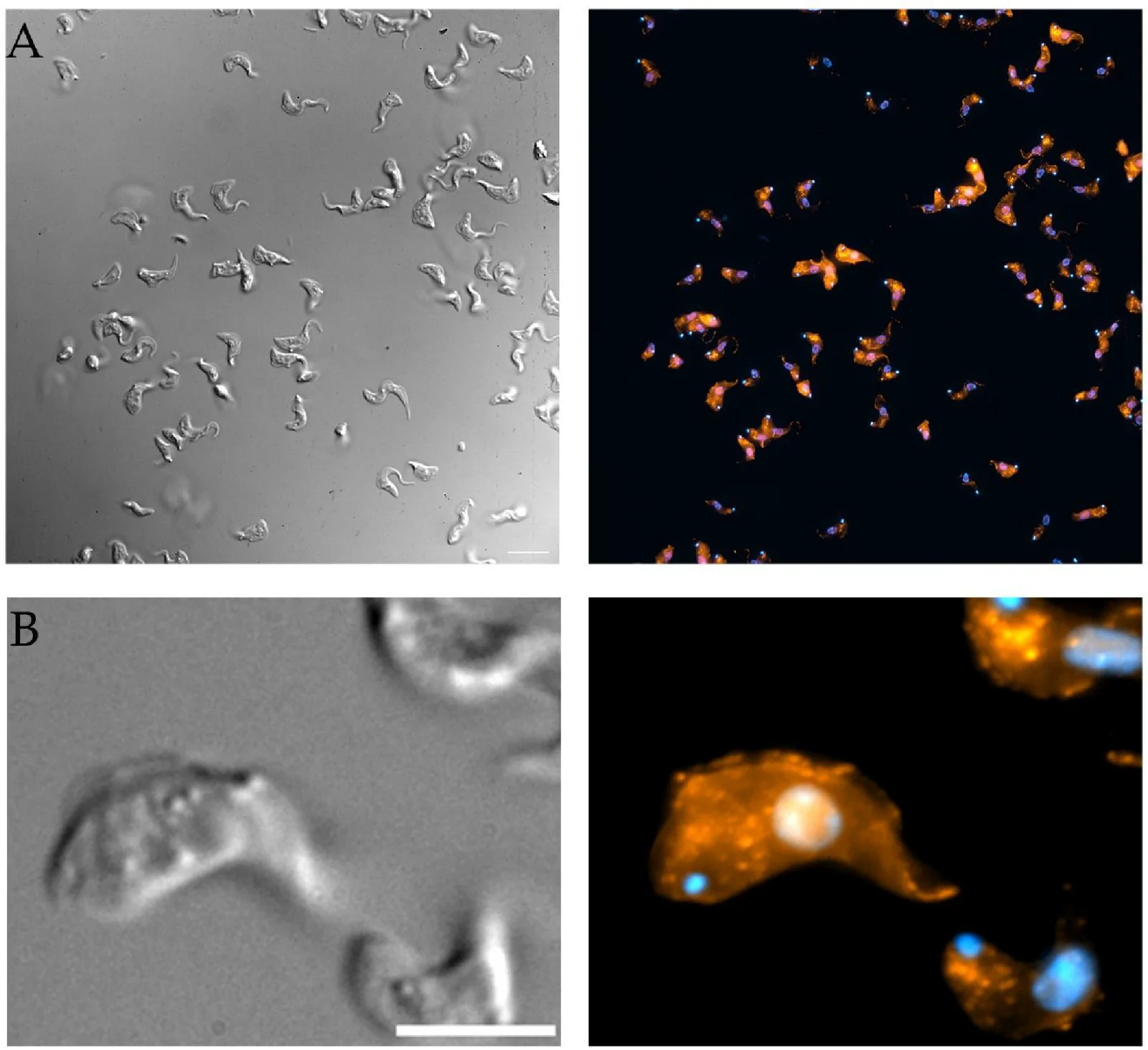
Fluorescence-activated cell sorting (FACS) was used to isolate stumpy cells of the tdTomato NLS-GFP:PAD1 3′UTR line, ensuring a pure stumpy population prior to infection. (**A**) Stumpy cells from an SIF-induced stumpy culture (grown to 5×10^5^ cells/ml and kept for 48 hr), after FACS to confirm sorting success. Cells were fixed in 4% paraformaldehyde (PFA) immediately after sorting, stained with DAPI (blue), and labelled with an anti-PAD1 antibody (orange); scalebar: 20 μm. (**B**) High-resolution immunofluorescence (IF) image of a single stumpy trypanosome displaying characteristic stumpy morphology and strong *pad1* signal (orange); scalebar: 10 μm.

Consistent with published data on stumpy infections (Peacock et al., 2012), male flies showed the highest infection rates (Figure 2B). Importantly, we found that non-teneral female flies can also be infected with slender cells. Midgut infections were observed in 9.5% of non-teneral female flies infected with slender cells and 5.6% with stumpy cells (Figure 2C). Although overall numbers were low, it is noteworthy that only slender cells caused any salivary gland infections in non-teneral female flies (Figure 2C). A table of all percentages of fly infections, replicates, and sex can be found in Supplementary file 1B.

We have previously shown that slender and stumpy forms both differentiate to procyclic forms with comparable kinetics (Schuster et al., 2021). To assess whether slender cells transcriptionally transition through a stumpy state or differentiate directly into the procyclic form, we performed RNA sequencing of slender and stumpy parasites throughout differentiation. For this, we used the same cell line as in Schuster et al., 2021, containing an EP1:YFP fusion protein and NLS-GFP:PAD1 3′UTR stumpy reporter. In vitro differentiation of both slender and stumpy cultures was induced by glucose depletion, addition of *cis*-aconitate, and temperature drop to 27°C (Mowatt and Clayton, 1987; Richardson et al., 1988; Roditi et al., 1989; Ziegelbauer et al., 1990; Matthews and Gull, 1994; Dean et al., 2009).

**Figure 3.**
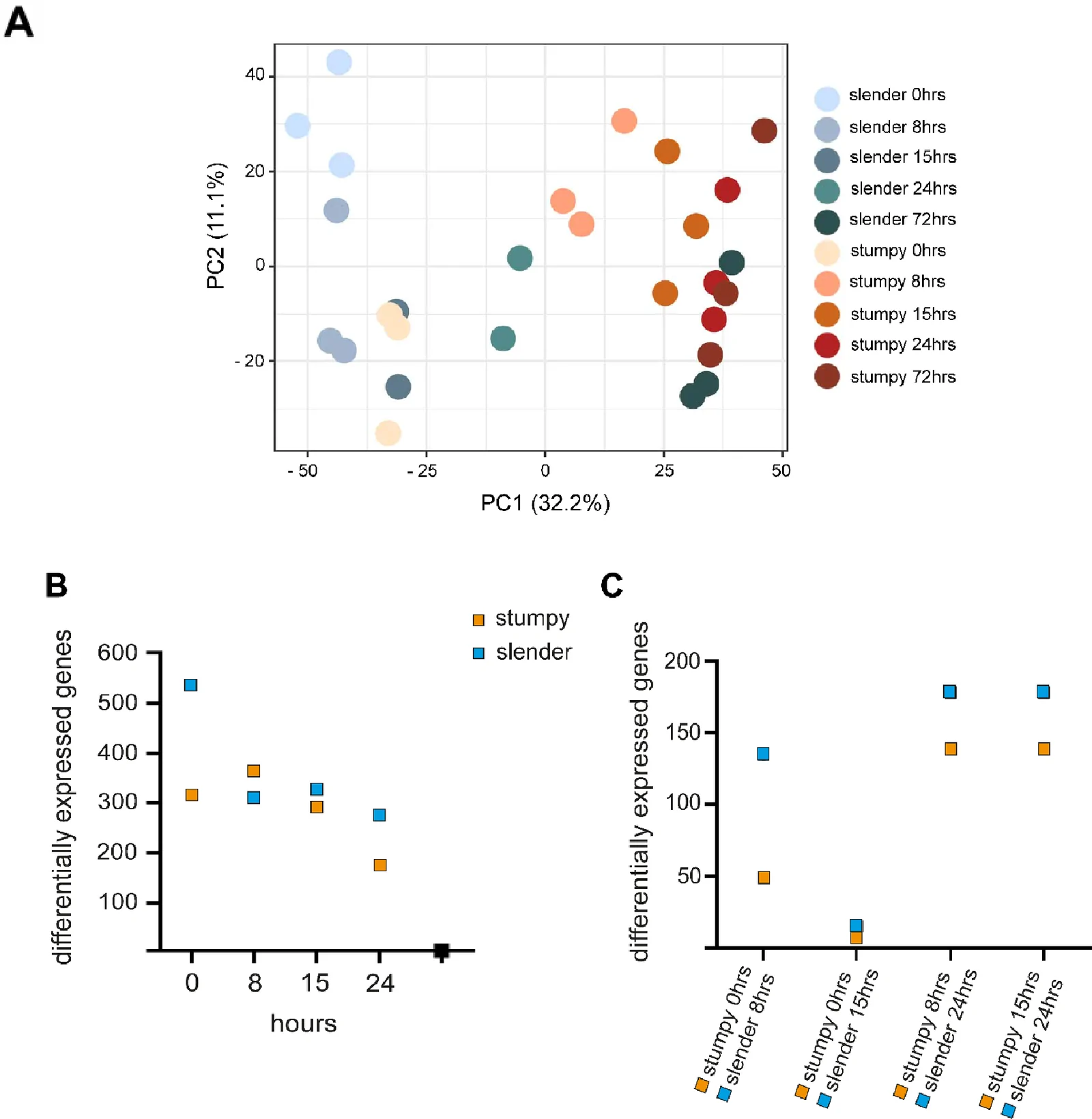
RNA sequencing of slender and stumpy trypanosomes differentiating into procyclic forms, performed in biological triplicates. (**A**) Principal component analysis (PCA) showing the transcriptional progression to procyclic forms for slender (blue/green) and stumpy (orange/red) cells. The trajectories remain distinct until converging at 72 hr. (**B**) Number of differentially expressed genes that are upregulated in slender compared to stumpy forms at corresponding time points during differentiation. Genes with an absolute log2FC>2 and an adjusted p-value<0.01 were classified as differentially expressed. Corresponding volcano plots and detailed gene counts can be found in Figure 3—figure supplement 1. (**C**) Differentially expressed genes that are upregulated between offset time points during slender and stumpy differentiation. The offset comparison aligns slender 15 hr with stumpy 0 hr, where only 22 genes are differentially expressed. Genes with an absolute log2FC>2 and an adjusted p-value<0.01 were classified as differentially expressed. Corresponding volcano plots and detailed gene counts can be found in Figure 3—figure supplement 2.

Slender or stumpy cells were collected in triplicates at five time points throughout differentiation, 0, 8, 15, 24, and 72 hr, and subjected to paired-end sequencing (Figure 3). Hierarchical clustering identified two biological replicates as outliers, which were excluded from further analysis.

**Figure 3—figure supplement 1.**
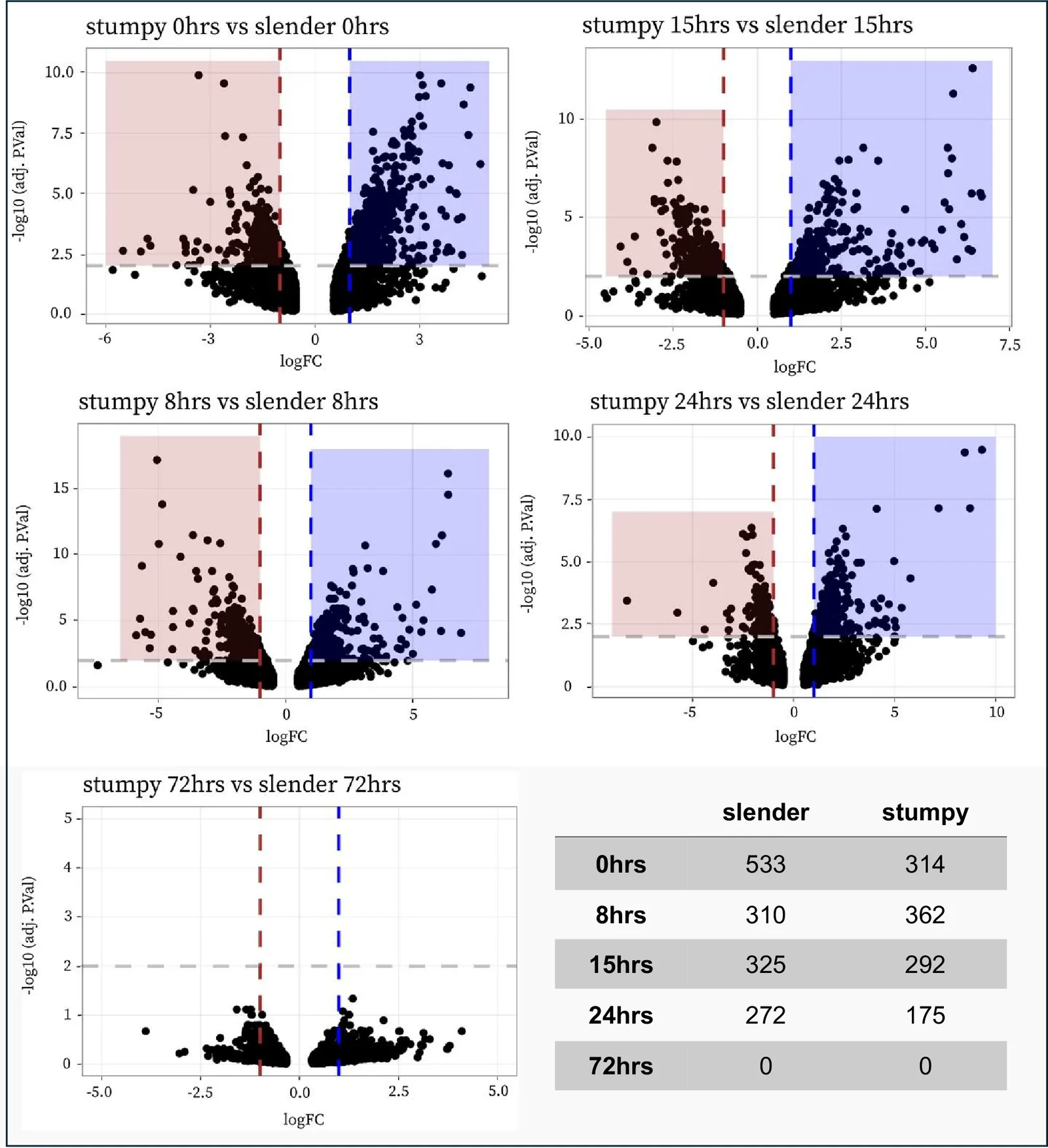
Volcano plots showing differential gene expression between stumpy (red) and slender (blue) *T. brucei* forms during in vitro differentiation to the procyclic form at 0, 8, 15, 24, and 72 hrs after induction. Differentiation was initiated by adding *cis*-aconitate, lowering the temperature to 27°C, and depleting glucose in the medium. Each black dot represents one gene. Genes within the coloured boxes show 2× upregulation in slender (blue dotted line) or 2× upregulation in stumpy (red dotted line) cells, with a p-value≤0.01 (grey dotted line). Red and blue boxes highlight significantly upregulated genes in stumpy or slender, respectively, with the exact gene counts listed in the accompanying table. logFC = log2 fold change; hrs = hours after the addition of *cis*-aconitate.

**Figure 3—figure supplement 2.**
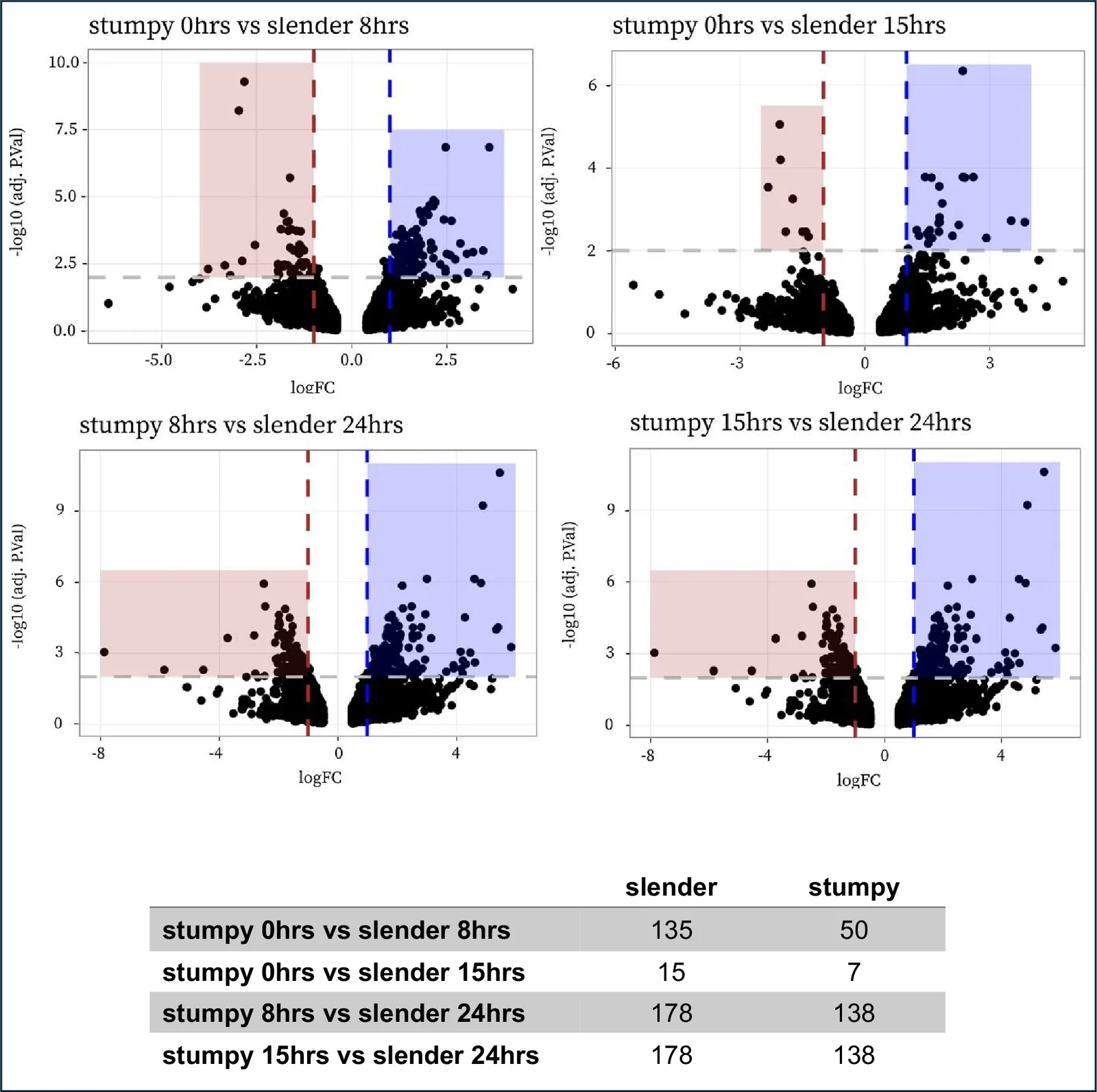
Volcano plots showing differential gene expression of stumpy (red) and slender (blue) *T. brucei* forms following in vitro differentiation to the procyclic form. Differentiation was initiated by adding *cis*-aconitate, lowering the temperature to 27°C, and depleting glucose. An offset comparison – based on proximity in the principal component analysis (PCA) plot (Figure 3A) – aligns slender cells at 15 hours (hrs) with stumpy cells at 0 hrs. Each black dot represents one gene. Genes within the coloured boxes show 2× upregulation for slender (blue dotted line) or 2× upregulation for stumpy (red dotted line) cells, with a p-value≤0.01 (grey dotted line). Red and blue boxes highlight significantly upregulated genes in stumpy or slender, respectively, with the exact numbers of differentially expressed genes listed in the accompanying table. Notably, slender cells at 15 hrs exhibit a similar gene expression profile to that of stumpy trypanosomes after 0 hrs, before diverging again at later time points. logFC = log2 fold change; hrs = hours after the addition of *cis*-aconitate.

**Figure 3—figure supplement 3.**
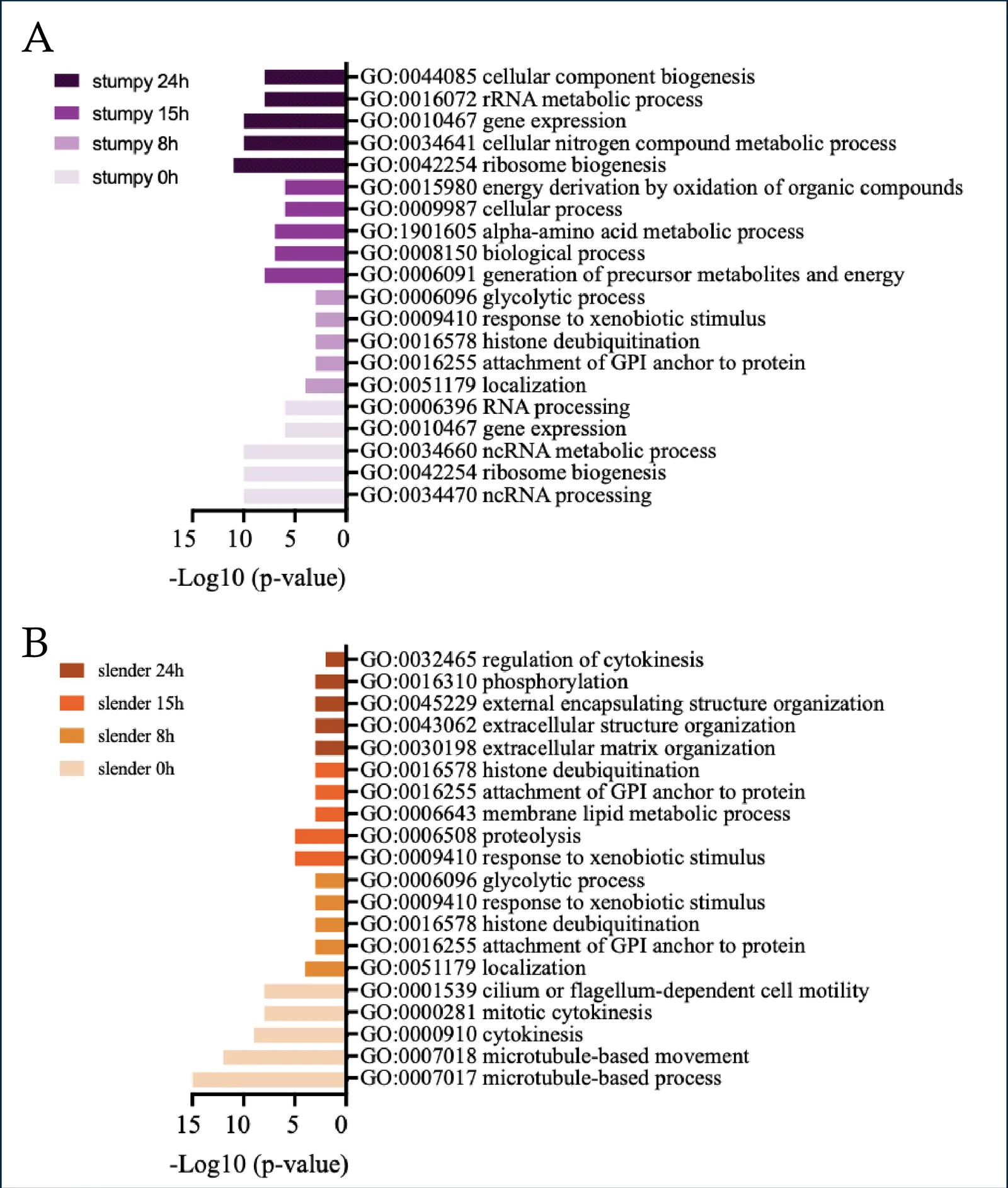
During differentiation into the procyclic form, slender and stumpy parasites exhibit gene expression profiles associated with distinct biological processes and molecular functions. Gene Ontology (GO) enrichment analysis between corresponding time points identified genes with a log2 fold change of at least 1 (indicating 2× expression) and a p-value of ≤0.01 for either slender (orange) or stumpy (purple) forms at 0, 8, 15, 24, and 72 hr (Figure 3—figure supplement 1). GO annotations were sourced from the TriTryp.org database (TriTryp.org) and refined using Revigo. The most significantly enriched GO terms for each time point are shown.

**Figure 3—figure supplement 4.**
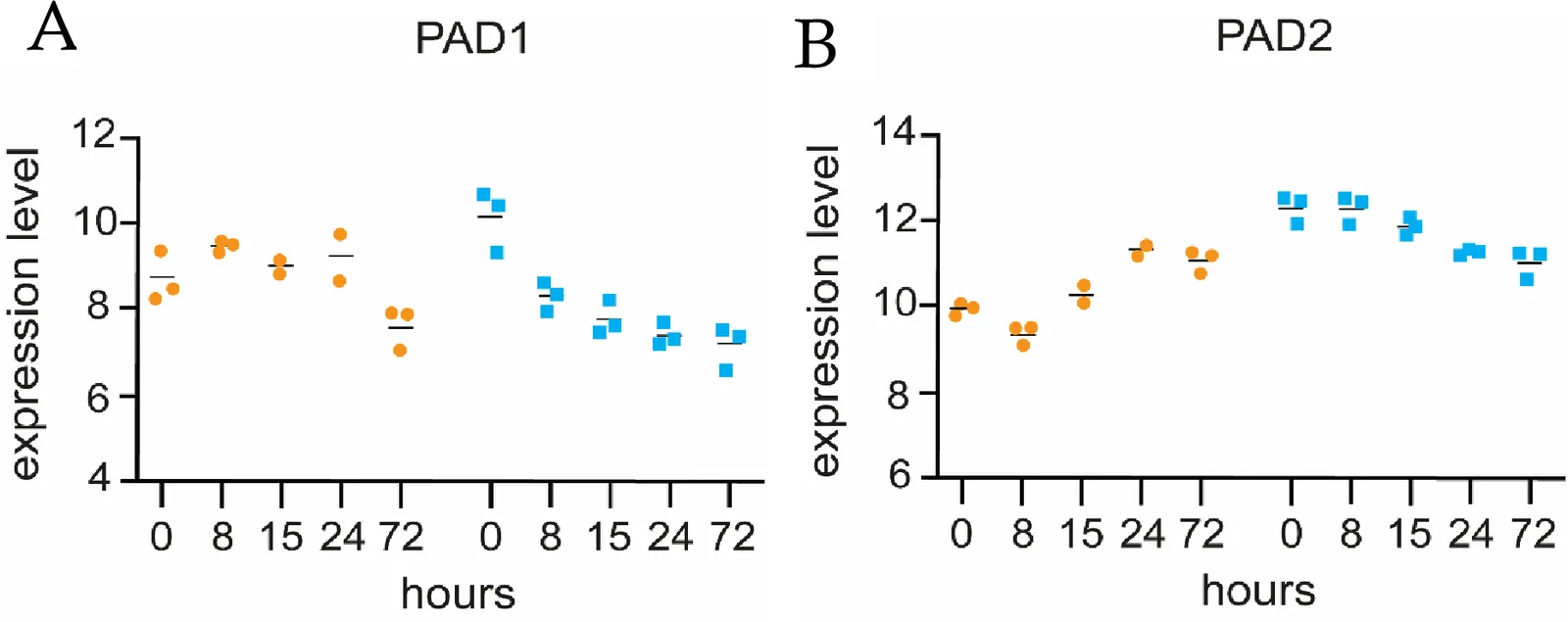
Dot plots showing expression levels of genes associated with the stumpy form – PAD1 (**A**) and PAD2 (**B**) – in slender (orange) and stumpy (blue) forms during in vitro differentiation. Differentiation was induced by the addition of *cis*-aconitate, reduction of temperature to 27°C, and glucose depletion. RNA sequencing was performed in triplicates; each coloured dot represents one replicate (1000 cells), and the black line indicates the mean log2 counts per million (CPM) value. A statistical significance assessment using Welch’s t-test showed no significant differences in PAD1 expression between stumpy and slender forms at 0 hr (p-value=0.059), nor in PAD1 expression trajectory between 0 to 72 hr (p-value=0.102). In contrast, PAD2 expression differed significantly between slender and stumpy forms at the baseline (0 hr; p-value<0.001) and showed significant changes in expression from 0 to 72 hr (p-value<0.003), suggesting differential regulation prior to the transition to the procyclic stage.

**Figure 3—figure supplement 5.**
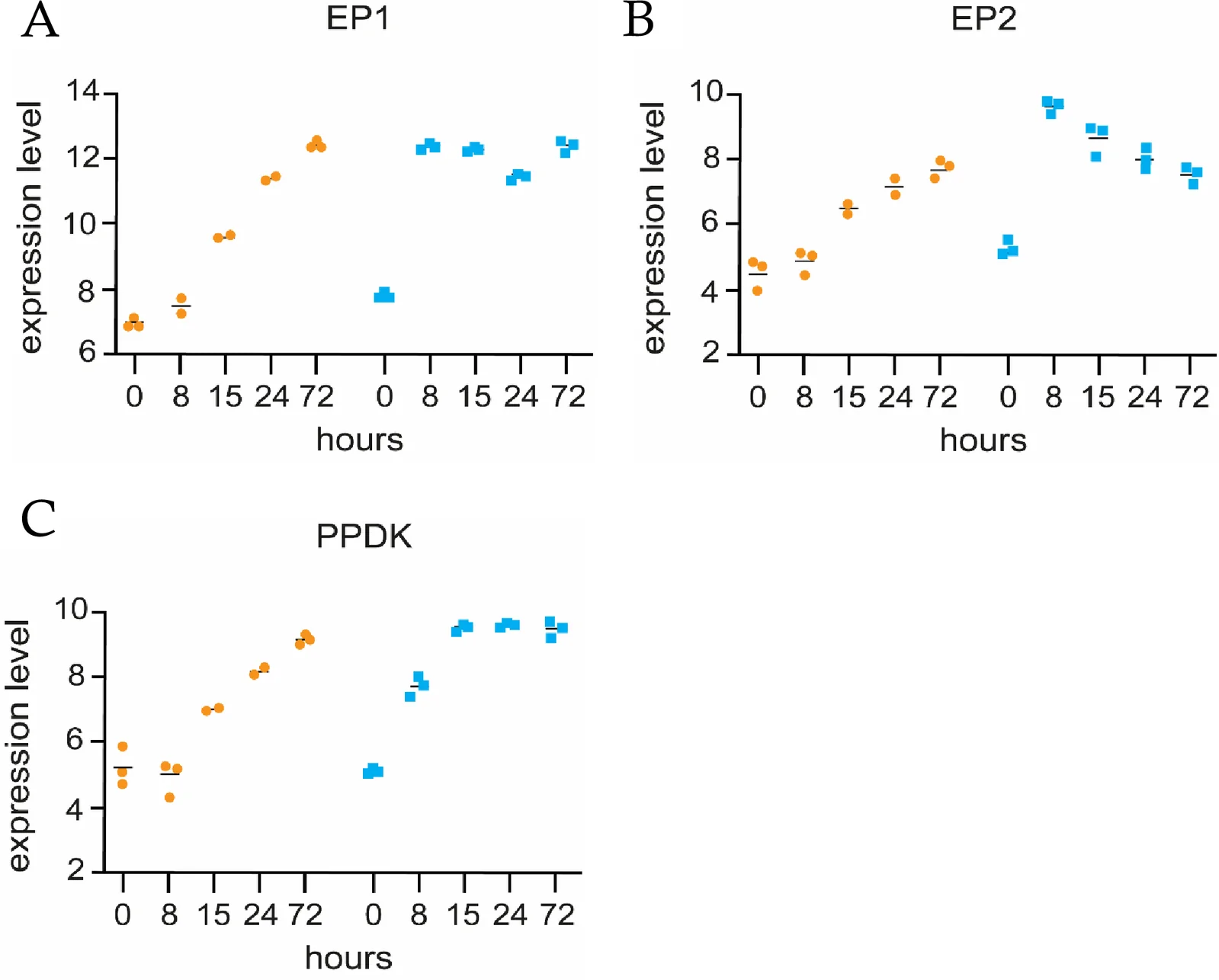
Dot plots showing expression levels of genes associated with the procyclic form – EP1 (**A**), EP2 (**B**), and pyruvate phosphate dikinase (PPDK) (**C**) – in slender (orange) and stumpy (blue) forms during in vitro differentiation. Differentiation was induced by the addition of *cis*-aconitate, reduction of temperature to 27°C, and glucose depletion. Stumpy forms reach procyclic-like expression levels by approximately 8 hr, whereas slender forms display a more gradual increase. RNA sequencing was performed in triplicates; each coloured dot represents one replicate (1000 cells), and the black line indicates the mean log2 counts per million (CPM) value.

Principal component analysis was performed, with the first principal component accounting for 32.2% and the second principal component for 11.1% of total variance (Figure 3A).

When comparing corresponding time points between slender and stumpy forms, e.g., slender 8 hr vs stumpy 8 hr, both cell types initially exhibit a high number of differentially expressed genes. This number gradually decreases, reaching zero at 72 hr (Figure 3B; Figure 3—figure supplement 1). Interestingly, when comparing all time points, an additional convergence with only 22 differentially expressed genes is observed between slender 15 hr and stumpy 0 hr (Figure 3C; Figure 3—figure supplement 2).

At first glance, this might suggest that slender cells transition into the stumpy form at this point before proceeding to differentiate into procyclic forms in the same way as stumpy cells. However, comparison of subsequent time points reveals a different pattern: at slender 24 hr and stumpy 8 hr, the number of differentially expressed genes increases markedly to 316. This elevated number remains relatively consistent until both cell types converge at 72 hr, where no differentially expressed genes are detected (Figure 3B; Figure 3—figure supplements 1 and 2).

In line with these findings, Gene Ontology (GO) enrichment analysis reveals that gene expression profiles of slender and stumpy cells throughout differentiation are associated with distinct molecular functions and biological processes. In slender cells, the most significant GO terms are related to extracellular structure and matrix organisation, glycolytic processes, and proteolysis (Figure 3—figure supplement 3). In contrast, stumpy cells show enrichment for genes that are involved in RNA processing, ribosome biogenesis, as well as cellular component biogenesis, consistent with them re-entering the cell cycle to become procyclic forms (Figure 3—figure supplement 3). Transcript data of the stumpy markers PAD1 and PAD2 show different expression levels for slender and stumpy forms, before reaching similar levels by 72 hr, when both forms had become procyclic (Figure 3—figure supplement 4). Genes associated with the procyclic form, such as the procyclin surface proteins EP1 and EP2, as well as pyruvate phosphate dikinase, start with low expression levels for both slender and stumpy forms. However, expression increases to procyclic levels by only 8 hr in stumpy cells but more gradually in slender cells, reaching comparable levels only by 72 hr (Figure 3—figure supplement 5).

**Figure 4.**
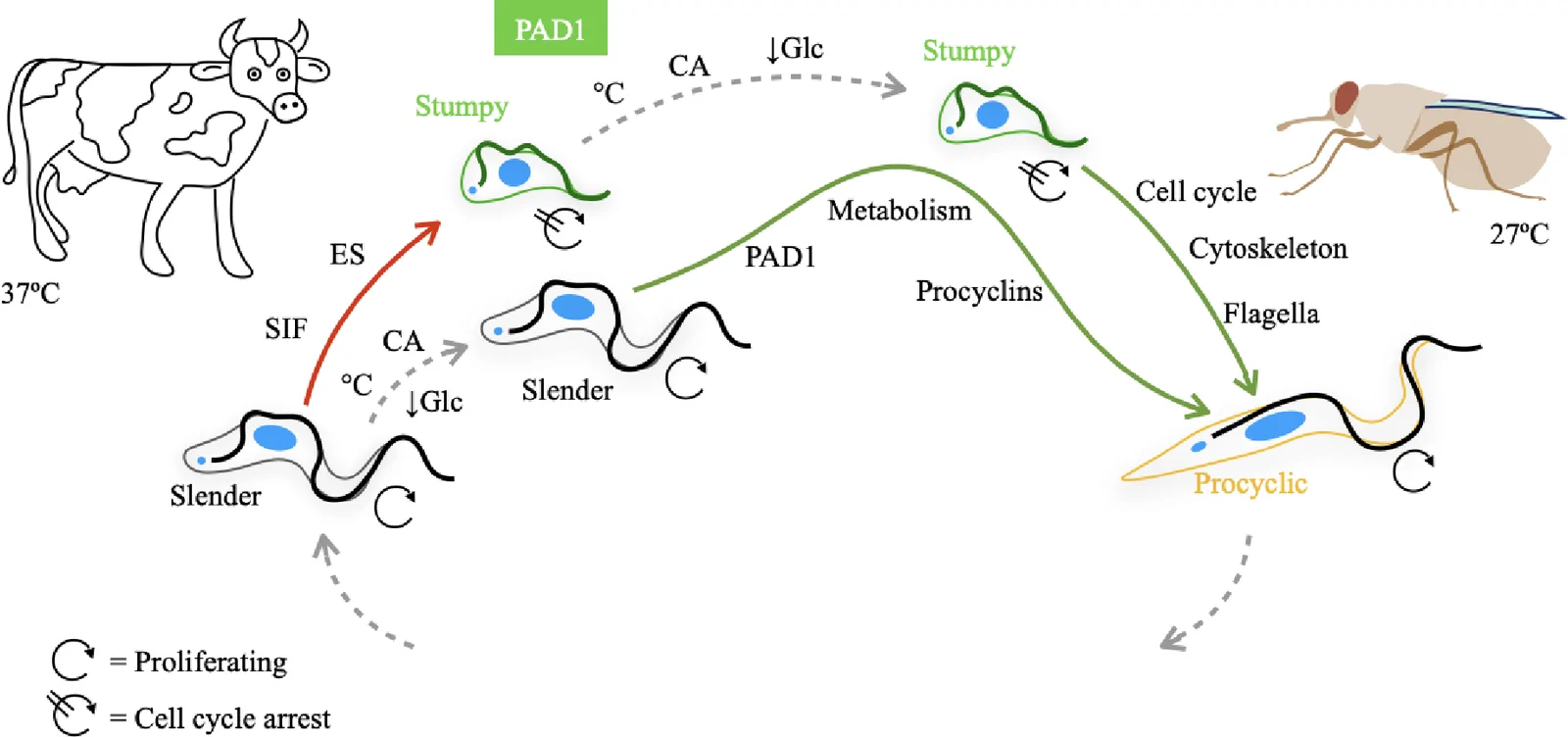
Slender and stumpy bloodstream forms must activate distinct pathways to transform into the procyclic form in the tsetse fly. Differentiation to the PAD1-positive (green), cell-cycle-arrested, short stumpy form can be triggered by either SIF or ES attenuation (Zimmermann et al., 2017). Stumpy forms have a 2- to 3-day window to be ingested by a tsetse fly before they perish. When a tsetse fly takes a bloodmeal, it can ingest both slender and stumpy forms. Once in the fly’s midgut, both forms begin their transformation into the procyclic fly form. During the initial 15 hr, slender forms shift towards stumpy gene expression before diverging again. Stumpy forms need to reactivate the cell cycle, fully switch to proline metabolism, and elongate both their cytoskeleton and flagella (Figure 3—figure supplement 3A). Slender forms must activate the essential PAD1 pathway, complete the switch to proline metabolism, and change to a procyclin coat (Figure 3—figure supplement 3B). By 72 hr into differentiation, both slender and stumpy forms have transitioned into the procyclic form. This figure was adapted from Schuster et al., 2021.

Collectively, these data indicate that differentiation in slender and stumpy trypanosomes proceeds via distinct gene expression programmes. Rather than following a shared trajectory, each form activates a unique set of genes to transition into the first fly form, the procyclic form (Figure 4).

## Discussion

Historically, the lack of molecular markers to distinguish slender and stumpy forms resulted in conflicting reports on their transmissibility to the tsetse fly. Early experiments indicated that higher trypanosome levels in mammalian blood correlated with more infected flies. Since stumpy forms arise through a density-dependent mechanism, increased bloodstream concentrations inevitably meant more stumpy forms in mammalian hosts, an uncontrollable factor in these early studies (Robertson, 1912; Hoof, 1947; Baker and Robertson, 1957; Wijers, 1958).

Moreover, the discovery of the secreted quorum sensing factor SIF in the 1990s reinforced the notion that this pathway serves to ensure the presence of stumpy forms (Reuner et al., 1997; Vassella et al., 1997). It was also shown that stumpy forms begin developing traits needed for survival in the fly midgut, such as an elaborated mitochondrion and expressing enzymes associated with the Krebs cycle (Vickerman, 1965; Brown et al., 1973; Hamm et al., 1990; Reuner et al., 1997). Previous research also suggested that the stumpy form was more adapted to life in the tsetse fly, pointing to their resistance to proteolytic stress and acidic conditions in vitro (Nolan et al., 2000). Later, however, it was shown that the tsetse midgut is actually an alkaline environment, making the stumpy resistance to acidic conditions mute in the case of midgut survival (Liniger et al., 2003).

Taken together, it is understandable why early studies concluded that only stumpy-form trypanosomes could infect tsetse flies. This assumption, however, gave rise to the transmission paradox: under conditions of low parasitaemia during chronic infections, how can sufficient numbers of stumpy forms be maintained to ensure parasite transmission and sustain the disease cycle?

The ability to study the differences between slender and stumpy cells changed upon the discovery of the first stumpy-specific molecular marker, the PAD1, in 2009 (Dean et al., 2009). Using the PAD1 marker, it was shown that skin-resident trypanosomes reveal a locally increased proportion of stumpy forms, providing a possible solution to this transmission paradox. In mice, the proportion of stumpy cells in the skin ranged from 8% to 80%, suggesting that these parasites might significantly contribute to infection dynamics (Capewell et al., 2016).

Our recent study using artificial human skin models revealed that trypanosomes freshly deposited into the skin by tsetse flies develop into a quiescent form that cannot reinfect the vector again (Reuter et al., 2023). Although this finding does not address parasites that migrate back into the skin from circulation, it highlights the complex dynamics occurring within tissue. Ultimately, the extent to which skin-resident populations contribute to tsetse transmission remains unclear, as direct quantification is currently not feasible.

In 2021, we offered an alternative explanation for the transmission paradox, namely that slender-form trypanosomes can also infect and complete the life cycle in tsetse flies. All bloodstream-form trypanosomes taken up in a bloodmeal have the potential to infect a mammalian host by establishing salivary gland infection in tsetse flies (Schuster et al., 2021). While all our data supported this conclusion, some questions were raised, which we have now answered.

To mimic more natural conditions, we conducted infection experiments using slender forms without immune-suppressors, both male and female flies, and non-teneral flies.

The definition of a non-teneral and teneral fly is of great importance here.

The age of a teneral fly ranges anywhere from 8 to 72 hpe, with the important criterion that they have not taken up any blood (Otieno et al., 1983; Walshe et al., 2011). Thus, when infecting teneral flies, their first bloodmeal is the infectious one. In contrast, we infected our non-teneral flies between 144 and 168 hpe, having received two regular bloodmeals, each 2 days apart, before the third infectious feeding.

While it could be argued that it would be better to use even older flies, the lifespan of the tsetse must be taken into consideration to ensure survival through the 30 days required for *T. brucei* to complete development in the vector. It has been suggested that tsetse flies in the wild may live longer than their laboratory-raised counterparts, though estimated and observed lifespans vary widely. The mean lifespan ranges from 35 to 178 days for female and only 21 to 28 days for male flies (Jackson, 1949; Welburn and Maudlin, 1999; Vale and Torr, 2005; Haines et al., 2020). Regardless of the individual lifespan of a fly, a trypanosome infection is permanent.

We have demonstrated, in a statistically well-controlled manner, that a single slender trypanosome is capable of infecting the tsetse fly. Here, we demonstrate that slender forms can establish infections without immunosuppressive treatment under the conditions tested. This infectivity was observed in both teneral and non-teneral, as well as in both male and female flies, indicating that slender forms retain transmission potential across different fly demographics.

The study by Ngoune et al., 2025, does not disprove this finding (Ngoune et al., 2025). In their infection experiments, only 63% of the ‘stumpy’ cells expressed PAD1, meaning that 37% must, by definition, have been slender forms. This heterogeneity complicates the interpretation of their results. It is also worth noting that Ngoune et al. used the AnTat 1.1E strain, which they maintained in culture without methylcellulose. We have observed that, during adaptation to methylcellulose-free medium, our bona fide pleomorphic AnTat 1.1 strain gradually loses its developmental competence. This could account for the heterogenous ‘stumpy’ population reported by Ngoune et al.

Moreover, more than 40% of the adult male flies in their study did not survive the 28-day period required before dissection, further complicating the interpretation of their infection data.

In response to our original work, Matthews and Larcombe postulated that ‘molecular characteristics’ (PAD1 in particular) define a stumpy cell and that the eponymous morphological changes (e.g. stumpy formation) are no longer valid criteria (Matthews and Larcombe, 2022). Although irreversible cell-cycle arrest is still an accepted hallmark of stumpy trypanosomes, we cannot exclude that in the tsetse fly, the dividing slender population arrests the cycle and then transitions to the procyclic form. This scenario is of some theoretical interest, especially in view of the ongoing discussion about shallow vs. deep cell-cycle arrest, e.g., in stem cell quiescence (Urbán and Cheung, 2021). Therefore, we decided to conduct an RNA sequencing time course that would compare the transcriptional landscape of slender and stumpy bloodstream populations during differentiation to the procyclic form (Figure 3). Using FACS, slender cultures were sorted to exclude any PAD1-positive cells, while stumpy cultures were sorted to include 100% PAD1-expressing cells.

At the outset, more than 300 genes were differentially expressed between the two bloodstream forms. However, after 72 hr under differentiation conditions, no significant differences in gene expression remained; both slender and stumpy cells had transitioned into procyclic forms. Importantly, stumpy forms appear transcriptionally primed for rapid progression, while slender cells activate a distinct and temporally delayed gene expression program (Figure 3—figure supplement 5).

The transient similarity observed at the specific time point, slender 15 hr and stumpy 0 hr, may reflect a brief convergence in gene expression, but is not indicative of a shared developmental pathway. Instead, the sustained differences in transcriptional profiles and enriched GO terms (Figure 3—figure supplements 1–3) argue for fundamentally different regulatory mechanisms underlying the commitment to procyclic differentiation.

These data argue against a linear progression from slender to stumpy to procyclic. Instead, they suggest that slender forms transiently activate a subset of genes also expressed in stumpy cells but then follow a distinct transcriptional trajectory before ultimately converging with the stumpy pathway at 72 hr, when both forms adopt a procyclic identity (Figure 3). Thus, slender and stumpy forms remain transcriptionally distinct for at least the first 24 hr of differentiation (Figure 3B). At this point, their transcriptomes do not correspond clearly to any of the three canonical forms – slender, stumpy, or procyclic. These findings demonstrate that slender forms can differentiate directly into procyclic forms without passing through a bona fide stumpy stage, i.e., without cell-cycle arrest.

If slender forms can adopt a distinct transcriptional trajectory towards the procyclic state in vitro, there is no reason to assume that *T. brucei* could not use both routes – via stumpy or directly from slender – in the tsetse fly as well.

Additionally, there is one more observation that needs to be taken into account. We have shown that even small numbers of monomorphic trypanosomes strain 427 – incapable of differentiating into stumpy forms – can establish stable midgut infections in tsetse flies, yet fail to progress to salivary gland colonisation (Schuster et al., 2021). These monomorphic cells do not respond to SIF (Reuner et al., 1997; Vassella et al., 1997) and fail to upregulate PAD1 (Dean et al., 2009), remaining locked in the proliferative slender bloodstream form. Thus, slender trypanosomes are indeed capable of infecting the tsetse midgut, even if they are monomorphic. The PAD pathway is not strictly required for colonisation of the midgut. However, activation of the PAD pathway is essential for the generation of procyclic trypanosomes that are competent to complete development and colonise the salivary glands.

As a concluding remark, our results clearly demonstrate that slender bloodstream forms can successfully establish infection in the tsetse fly, thereby extending the conclusion of Larcombe et al., 2023, that transmission during high parasitaemia is predominantly mediated by stumpy and pre-committed slender intermediates (Larcombe et al., 2023).

Notably, according to criteria summarised in Larcombe et al.’s own developmental commitment classification (Larcombe et al., 2023; Figure 4A), the population we have used would be defined as ‘true slender’ – cells that keep their replicative competence and do not yet express PAD1 nor show stumpy morphology. In our infection experiments, these sorted slender cells continued active division at the time of fly infection (Figure 2—figure supplement 1C), confirming their identity as true slender forms under Larcombe et al.’s definition.

This alignment underscores that our transmission data were generated with the very cell type previously proposed to be both rare and transmission-incompetent.

Consequently, our findings resolve the apparent transmission paradox: while stumpy and intermediate forms dominate at high parasitaemia (Larcombe et al., 2023), true slender cells prevail during low-parasitaemia phases and are themselves sufficient to maintain the *T. brucei* life cycle through transmission. Thus, replicative slender form should be recognised as a bona fide transmission stage, not merely a proliferative bloodstream-form extreme. Therefore, in retrospect, our results are perhaps less unexpected than initially assumed.

## Materials and methods

### Cell line

The pleomorphic *T. brucei brucei* strain EATRO 1125 (serodome AnTat 1.1) (Le Ray et al., 1977) with an NLS-GFP PAD1 3′UTR molecular marker and an additional tdTomato fluorescence sequence (Reuter et al., 2023) was used for infection experiments and cultured as previously described (Schuster et al., 2021). For RNA sequencing experiments, the same cell line was used with an additional EP1:YFP fusion protein (Schuster et al., 2021). The trypanosome cell lines have been authenticated by RNA sequencing and the VSG expressed (serodome AnTat 1.1) was confirmed by antibody binding (immunofluorescence and western blotting). The cell lines tested negative for mycoplasma contamination. We have not used any cell lines from the list of commonly misidentified cell lines maintained by the International Cell Line Authentication Committee.

### Fly infection

Tsetse flies of the species *Glossina morsitans morsitans* were kept as previously described (Schuster et al., 2021).

Teneral flies received their first, and infectious, bloodmeal 24–72 hpe. They were infected with four trypanosomes per bloodmeal, either untreated or supplemented with 60 mM NAG.

Non-teneral flies (144–168 hpe) were given two non-infectious bloodmeals, each 2 days apart, prior to the infectious bloodmeal containing 1×10^6^ cells/ml of either slender or stumpy parasites. Prior to infection, FACS was performed to ensure 100% slender or stumpy population.

### Immunofluorescence

Cells were prepared, fixed, and stained as previously described (Schuster et al., 2021).

### Fluorescence-activated cell sorting

Cell sorting was performed using the FACSAria III (BD Biosciences, Franklin Lakes, NJ, USA). Cells were harvested, resuspended in pre-warmed TDB at 1×10^7^ cells/ml, transferred to FACS tubes through a 35 µm cell strainer cap. Cells were first gated based on the tdTomato signal to exclude debris and residual medium. Subsequently, GFP fluorescence from the NLS-GFP PAD1 3′UTR reporter was analysed. Depending on the desired population, gates were set to isolate either PAD1-positive (stumpy) or PAD1-negative (slender) cells with high purity. For each experiment, 1×10^6^ cells were sorted using a 100 µm nozzle at the lowest sorting speed to minimise mechanical stress. Sorted cells were collected into 15 ml tubes containing pre-warmed FCS, resulting in a final FCS concentration of 15% (vol/vol). After sorting, cell motility, concentration, and PAD1 signal were checked by microscopy.

### Bulk RNA sequencing

Both slender and stumpy trypanosomes were differentiated into procyclic forms as previously described. The 0 hr time point was collected immediately before differentiation was induced, and additional samples were taken at 8, 15, 24, 72 hr following addition of *cis*-aconitate.

Cells were harvested from biological triplicates, resuspended in 1 ml pre-warmed PBS containing 10% FCS, and transported to the Helmholtz Institute for RNA-based Infection Research (HIRI), Würzburg, in a 37°C incubation chamber.

Upon arrival, cells were washed twice with pre-warmed PBS to remove FCS in 1 ml of pre-warmed PBS 15 min prior to sorting. Calcein-AM Violet was added at a final concentration of 1 µM to label viable cells.

Live cells were sorted using an FACSAria III cytometer (BD Biosciences). 1000 cells per replicate were deposited into single wells of a 48-well plate containing 2.6 µl of 1× lysis buffer (Takara) and 0.01 µl of RNase inhibitor (40 U/µl; Takara). Sorting was performed in triplicates for each time point, and plates were immediately placed on ice and stored at –80°C.

Library preparation and sequencing were performed as previously described (Müller et al., 2018). Very briefly, lysates were supplemented with 0.2 µl ERCC Spike-in Control Mix 1 (Thermo Fisher Scientific) at a 1:20,000,000 dilution. Libraries were prepared using the SMART-Seq v.4 Ultra Low Input RNA Kit (Takara), utilising a quarter of the recommended reagent volumes. PCR amplification was performed for 27 cycles, and cDNA was purified with Agencourt AMPure XP beads (Beckman Coulter) and 15 µl elution buffer (Takara). Library quantification was carried out using a Qubit 3 Fluorometer and dsDNA Hs Assay Kit (Life Technologies), while quality assessment was performed using a 2100 Bioanalyzer with High Sensitivity DNA Kit (Agilent). 0.5 ng of cDNA was used as input for the Nextera XT (Illumina) tagmentation-based library preparation protocol. The reaction was performed at one quarter of the recommended volumes, with a 10 min tagmentation step at 55°C, and a 1 min extension step during PCR. Libraries were pooled and sequenced in paired-end mode with 2×75 cycles using Illumina NextSeq 500.

### Analysis of bulk RNA sequencing data

Bulk RNA sequencing analysis was performed according to scripts and online resources published by Berry et al., 2021, using RStudio (version 4.02). Read demultiplexing and quality control were performed with FastQC (version 0.12.1, Andrews, 2012). Adaptors were removed and reads were trimmed using Fastp (version 0.23.4, Chen, 2023).

The *T. b. brucei* TREU 972 reference genome was obtained from the Tritryp database (TritrypDB). rRNA was removed (Aslett et al., 2010), and to remove redundancy, only one representative from each gene group was retained, while others were masked. Gene groups were defined based on sequence similarity using CD-HIT software (Li and Godzik, 2006).

Kallisto was used for read alignment, and genes with ≥3 counts per million (cpm) in at least two samples were retained for downstream analysis. Data normalisation was performed using the EdgeR package in RStudio (Robinson et al., 2010; R Development Core Team, 2021).

SL152 (slender 15 hr, second replicate) and SL241 (24 hr, first replicate) were considered outliers based on hierarchical clustering distance using the Minkowski metric (Lee and Willcox, 2014).

Differentially expressed genes were identified using DESeq2, EdgeR, and Limma packages in RStudio (Love et al., 2014; Ritchie et al., 2015; Robinson et al., 2010; R Development Core Team, 2020), applying significance criteria of log2 fold change >1 and p-value<0.01.

### Statistics

Two-tailed Fisher’s exact tests were performed for all fly infection data using GraphPad Prism version 9.0.0 for Windows (GraphPad software, San Diego, CA, USA, http://www.graphpad.com).

Welch’s t-test was performed for all gene expression data using RStudio (version 4.02) and the tidyverse 2.0.0 package.

## Data Availability

All data generated or analyzed during this study are included in the manuscript and supporting files. The RNA sequence data have been published under NCBI GEO accession number GSE316496. The following dataset was generated: LisackJN KreisA HaufL KrenzerJ ImdahlF EngstlerM 2026Research advance on unexpected plasticity in the life cycle of *Trypanosoma brucei*NCBI Gene Expression OmnibusGSE316496

## Additional files

MDAR checklist

Supplementary file 1. Absolute numbers of tsetse fly infections using slender bloodstream forms of *T. brucei*. (**A**) Absolute numbers of tsetse fly infections using slender bloodstream forms of *T. brucei*, with or without the addition of *N*-acetylglucosamine (NAG). Both male and female flies were infected with blood containing 200 slender cells per ml of blood, either untreated or supplemented with the immune-suppressing chemical, NAG (60 mM). Tsetse flies have an estimated drinking volume of 20 µl (Gibson and Bailey, 2003), which results in an uptake of 4 parasites per bloodmeal. All flies were dissected 35 days post infection, and their midgut (MG), proventriculus (PV), and salivary glands (SG) examined for parasite presence. Prior to infection, slender cells of the tdTomato NLS-GFP:PAD1 3′UTR line were verified to lack *pad1* expression, confirming pure slender identity (<0.05% PAD1 positive). (**B**) Absolute numbers of infections in non-teneral tsetse flies with either slender or stumpy *T. brucei* cells. Male and female flies were infected with untreated blood containing 1×10^6^ cells/ml. The tdTomato NLS-GFP:PAD1 3′UTR cell line enabled FACS-based separation of stumpy (PAD1 positive, GFP in nucleus) and slender (PAD1 negative, no nuclear fluorescence) forms prior to infection. All non-teneral flies were 144–168 hr post eclosion (hpe) and had already received two non-infectious bloodmeals prior to the infectious feed. Flies were dissected 35 days post infection, and their midgut (MG), proventriculus (PV), and salivary glands (SG) examined for parasite presence.
