# Supplementary material for: Replicative slender bloodstream forms complete transmission of Trypanosoma brucei without prior differentiation into stumpy forms
Source: eLife. 2026 Aug 19;14:RP108688. doi: 10.7554/eLife.108688 (PMC13489625; doi:10.7554/eLife.108688)
Supplement: Supplementary file 2 [file elife-108688-supp1.docx]

| No. of flies infected | Fly sex | No. of slender cells/ml | Concentration of NAG | Days until dissection | No. of flies dissected | MG | PV | SG |
| --- | --- | --- | --- | --- | --- | --- | --- | --- |
| 37 | male | 200 | 0 mM | 35 | 16 | 0 | 0 | 0 |
| 19 | female | 200 | 0 mM | 35 | 17 | 1 | 1 | 0 |
| 24 | male | 200 | 0 mM | 35 | 24 | 5 | 4 | 1 |
| 24 | female | 200 | 0 mM | 35 | 22 | 2 | 2 | 0 |
| 24 | male | 200 | 0 mM | 35 | 21 | 1 | 1 | 0 |
| 9 | female | 200 | 0 mM | 35 | 9 | 1 | 1 | 0 |

| No. of flies infected | Fly sex | No. of slender cells/ml | Concentration of NAG | Days until dissection | No. of flies dissected | MG | PV | SG |
| --- | --- | --- | --- | --- | --- | --- | --- | --- |
| 37 | male | 200 | 60 mM | 35 | 35 | 3 | 3 | 2 |
| 19 | female | 200 | 60 mM | 35 | 18 | 1 | 1 | 0 |
| 26 | male | 200 | 60 mM | 35 | 24 | 1 | 1 | 1 |
| 27 | female | 200 | 60 mM | 35 | 27 | 1 | 1 | 0 |
| 26 | male | 200 | 60 mM | 35 | 26 | 7 | 4 | 2 |
| 9 | female | 200 | 60 mM | 35 | 8 | 3 | 3 | 0 |

Table A: Absolute numbers of tsetse fly infections using slender bloodstream forms of *T. brucei*, with or without the addition of *N*-Acetyl-Glucosamine (NAG). Both, male and female flies were infected with blood containing 200 slender cells per ml of blood, either untreated or supplemented with the immune-suppressing chemical, *N*-Acetyl-Glucosamine (NAG, 60mM). Tsetse flies have an estimated drinking volume of 20 µl (Gibson & Bailey, 2003), which results in an uptake of 4 parasites per bloodmeal. All flies were dissected 35 days post infection, and their midgut (MG), proventriculus (PV), and salivary glands (SG) examined for parasite presence. Prior to infection, slender cells of the tdTomato NLS-GFP:PAD1 3´UTR line were verified to lack *pad1* expression, confirming pure slender identity (< 0.05% PAD1 positive).

| No. of flies infected | Fly sex | No. of slender cells/ml | Concentration of NAG | Days until dissection | infectious feed | | No. of flies dissected | MG | PV | SG |
| --- | --- | --- | --- | --- | --- | --- | --- | --- | --- | --- |
| 20 | male | 1x10^6^ | 0 mM | 30 | 3^rd^ | 17 | | 0 | 0 | 0 |
| 20 | male | 1x10^6^ | 0 mM | 30 | 3^rd^ | 19 | | 2 | 2 | 2 |
| 21 | male | 1x10^6^ | 0 mM | 35 | 3^rd^ | 21 | | 5 | 4 | 2 |
| 20 | male | 1x10^6^ | 0 mM | 35 | 3^rd^ | 19 | | 0 | 0 | 0 |
| 20 | female | 1x10^6^ | 0 mM | 30 | 3^rd^ | 20 | | 1 | 0 | 0 |
| 21 | female | 1x10^6^ | 0 mM | 30 | 3^rd^ | 21 | | 2 | 2 | 2 |
| 20 | female | 1x10^6^ | 0 mM | 35 | 3^rd^ | 20 | | 0 | 0 | 0 |
| 20 | female | 1x10^6^ | 0 mM | 35 | 3^rd^ | 20 | | 0 | 0 | 0 |

| No. of flies infected | Fly sex | No. of stumpy cells/ml | Concentration of NAG | Days until dissection | infectious feed | No. of flies dissected | MG | PV | SG |
| --- | --- | --- | --- | --- | --- | --- | --- | --- | --- |
| 20 | male | 1x10^6^ | 0 mM | 38 | 3^rd^ | 20 | 2 | 2 | 1 |
| 21 | male | 1x10^6^ | 0 mM | 36 | 3^rd^ | 16 | 1 | 1 | 1 |
| 23 | male | 1x10^6^ | 0 mM | 35 | 3^rd^ | 19 | 2 | 2 | 0 |
| 14 | male | 1x10^6^ | 0 mM | 35 | 3^rd^ | 11 | 0 | 0 | 0 |
| 20 | female | 1x10^6^ | 0 mM | 38 | 3^rd^ | 19 | 1 | 0 | 0 |
| 20 | female | 1x10^6^ | 0 mM | 36 | 3^rd^ | 20 | 1 | 0 | 0 |
| 23 | female | 1x10^6^ | 0 mM | 35 | 3^rd^ | 19 | 0 | 0 | 0 |
| 15 | female | 1x10^6^ | 0 mM | 35 | 3^rd^ | 13 | 2 | 1 | 0 |

Table B: Absolute numbers of infections in non-teneral tsetse flies with either slender or stumpy *T. brucei* cells. Male and female flies were infected with untreated blood containing 1x10^6^ cells/ml. The tdTomato NLS-GFP:PAD1 3´UTR cell line enabled FACS-based separation of stumpy (PAD1 positive*,* GFP in nucleus) and slender (PAD1 negative, no nuclear fluorescence) forms prior to infection. All non-teneral flies were 144-168 hours post eclosion (hpe) and had already received two non-infectious bloodmeals prior to the infectious feed. Flies were dissected 35 days post infection, and their midgut (MG), proventriculus (PV), and salivary glands (SG) examined for parasite presence.
