# Supplementary material for: Replicative slender bloodstream forms complete transmission of Trypanosoma brucei without prior differentiation into stumpy forms
Source: eLife. 2026 Aug 19;14:RP108688. doi: 10.7554/eLife.108688 (PMC13489625; doi:10.7554/eLife.108688)
Supplement: Peer review history

# Editor report: eLife Assessment

**DOI:** 10.7554/eLife.108688.4.sa0

**Authors:** Christine Clayton

**Affiliations:** Centre for Molecular Biology of Heidelberg University (ZMBH) Germany

**Keywords:** Fundamental, Solid

The manuscript concerns a **fundamental** and controversial question in *Trypanosoma brucei* biology and the parasite life cycle, whether or not dividing slender bloodstream forms must transition to growth-arrested stumpy forms before differentiating to the procyclic form in the Tsetse midgut. The authors provide further evidence that slender bloodstream forms can infect Tsetse flies, and that although their differentiation is considerably delayed, they do not become classical stumpy forms during the process. The study is **solid** in design and execution, and addresses several criticisms made of the authors' earlier work, although discrepancies with results from other laboratories remain.

# Referee report: Reviewer #2 (Public review)

**DOI:** 10.7554/eLife.108688.4.sa1

[Editors' note: this version has been assessed by the Reviewing Editor without further input from the original reviewers.]

Summary:

This paper is an exciting follow-up to two recent publications in eLife: one from the same lab, reporting that slender forms can successfully infect tsetse flies (Schuster, S et al., 2021), and another independent study claiming the opposite (Ngoune, TMJ et al., 2025). Here, the authors address four criticisms raised against their original work: the influence of N-acetyl-glucosamine (NAG), the use of teneral and male flies, and whether slender forms bypass the stumpy stage before becoming procyclic forms.

Strengths:

We applaud the authors' efforts in undertaking these experiments and contributing to a better understanding of the *T. brucei* life cycle. The paper is well-written and the figures are clear.

Comments on revisions:

We thank the authors for the revised manuscript and for considering our comments.

# Author comment: Author response

**DOI:** 10.7554/eLife.108688.4.sa2

**Authors:** Markus Engstler, Carina Praisler, Jaime Lisack, Anna Sophie Kreis, Laura Hauf, Johanna Odenwald, Fabian Imdahl

**Affiliations:** University of Würzburg Würzburg Germany; University of Würzburg Würzburg Germany; University of Würzburg Würzburg Germany; University of Würzburg Würzburg Germany; University of Würzburg Würzburg Germany; University of Würzburg Würzburg Germany; Helmholtz Institute for RNA-based Infection Research Würzburg Germany

The following is the authors’ response to the previous reviews

**Public Reviews:**

**Reviewer #2 (Public review):**

Summary:

This paper is an exciting follow-up to two recent publications in eLife: one from the same lab, reporting that slender forms can successfully infect tsetse flies (Schuster, S et al., 2021), and another independent study claiming the opposite (Ngoune, TMJ et al., 2025). Here, the authors address four criticisms raised against their original work: the influence of N-acetyl-glucosamine (NAG), the use of teneral and male flies, and whether slender forms bypass the stumpy stage before becoming procyclic forms.

Strengths:

We applaud the authors' efforts in undertaking these experiments and contributing to a better understanding of the *T. brucei* life cycle. The paper is well-written and the figures are clear.

Comments on revisions:

We thank the authors for the revised manuscript and for considering our comments.

We outline below the 3 points that, in our opinion, remain to be clarified.

(1) Effect of NAG on slender-form infections in tsetse flies

The conclusion that "NAG has a negligible effect on slender infections in tsetse flies" based on Figure 1, cannot be fully supported in the absence of a positive control. A relevant positive control is well established in the literature, namely that NAG promotes Tsetse infection by stumpy forms. Without such a control, it is not possible to exclude technical issues (for example, an ineffective NAG treatment), which would yield results similar to those presented in Figure 1.

We agree that an internal stumpy-form positive control would provide an additional technical reference. However, the enhancing effect of NAG on stumpy-form midgut infections is well established and was also demonstrated under the experimental framework of our original study (Schuster et al. 2021, Figure 2A).

The purpose of the present Research Advance was therefore not to re-establish the known effect of NAG on stumpy infections, but to test whether slender-form infections require NAG supplementation. Under the conditions tested here, slender bloodstream forms established midgut, proventriculus and salivary-gland infections also in the absence of NAG. We have revised the text accordingly to avoid implying a general absence of NAG effects and to make clear that our conclusion is restricted to slender-form infections under the conditions tested (line 128).

(2) Infection of non-teneral flies

Because the experiments shown in Figure 1 (teneral flies) and Figure 2 (non-teneral flies) were not conducted in parallel or under identical conditions, it is important that the figure legends clearly state the parasite numbers used in each case. Specifically, infections of teneral flies were performed with 200 parasites/mL (approximately 4 parasites per bloodmeal), whereas non-teneral infections used 1 × 10^6^ parasites/mL (approximately 20,000 parasites per bloodmeal?). At present, this information is scattered across the Methods and Supplementary Tables 1 and 2, making it difficult for readers to immediately appreciate that the parasite load differs by roughly 5,000-fold between these conditions.

As previously shown by the authors (Schuster et al., 2021) and in the Rotureau laboratory (Tsagmo Ngoune et al.), and as generally expected, the initial parasite dose strongly influences infection outcomes in teneral flies. In this context, it would be informative to know whether the authors have attempted infections of non-teneral flies using lower parasite numbers (noting that Tsagmo Ngoune et al. used a maximum of 10,000 parasites) and what the infection rate was.

Relatedly, the statement in line 370 appears to be an overgeneralization, as fly age was not directly tested under matched experimental conditions:

Line 370 - "Here, we unambiguously show that, in the absence of immunosuppressive treatment, slender forms can establish infections in tsetse flies, irrespective of the fly's age or sex."

We thank the reviewer for highlighting the inconsistent presentation of parasite doses between Figure 1 and 2. We agree this is confusing and have revised the figure legends to clearly state both the parasite concentration (cells/mL) and estimated fly uptake per bloodmeal for each experiment (Lines 143 and 206).

Regarding experiments with non-teneral flies using lower parasite numbers: We have not tested intermediate doses (e.g., 10,000 parasites/bloodmeal as used by Ngoune et al.) in non-teneral flies. Given that teneral flies already show relatively low infection rates even under optimal conditions, we chose the higher parasite dose (20,000 parasites/bloodmeal) for non-teneral flies to ensure sufficient statistical power for meaningful analysis of infection outcomes across different fly compartments.

We acknowledge the reviewer's concern regarding the statement in line 370 and have revised this sentence (line 375) to more accurately reflect our experimental conditions, avoiding overgeneralization beyond the specific parameters tested.

This reads now: “Here, we demonstrate that slender forms can establish infections without immunosuppressive treatment under the conditions tested. This infectivity was observed in both teneral and non-teneral, as well as in both male and female flies, indicating that slender forms retain transmission potential across different fly demographics. However, direct age comparisons under identical parasite doses remain to be tested.”

(3) Transcriptomic analysis

Supplementary Figure 8 lacks statistical analysis, which limits its interpretability. Two types of comparisons would be particularly helpful:

(i) a comparison of PAD1/2 expression levels between slender and stumpy forms at 0 h; and

(ii) for each gene, a comparison of the overall change in expression (from 0 to 72 h) between infections initiated with slender versus stumpy forms.

In addition, the figure legend should clarify what "expression levels" refer to. TPM? Normalized counts?

We appreciate this helpful comment and included statistical analysis for the expression of PAD1 and PAD2 (Supplementary Figure 8) between the two forms for the baseline (0 h) as well as during the differentiation to procyclic forms (0 h to 72 h) by using Welch´s t-test.

While PAD1 did not show a statistically significant difference in this analysis, PAD2 displayed significant differences in expression dynamics over time. This supports the broader transcriptomic observation that slender- and stumpy-initiated differentiation follow distinct transcriptional trajectories before converging at the procyclic stage.

We also clarified the figure legends showing the mean log2 counts per million (CPM) values.

Finally, for the benefit of the field, eLife could encourage publishing a collaborative study in which the Engstler and Rotureau laboratories exchange parasite lines and culture protocols (including media with and without methylcellulose) and perform tsetse fly infections in parallel in their respective laboratories. Such an approach could help resolve the remaining discrepancies and provide a valuable reference for the community.

We appreciate this constructive suggestion. A collaborative inter-laboratory study in which parasite lines, culture conditions and infection protocols are exchanged between the Engstler and Rotureau laboratories would be a valuable way to address the remaining discrepancies in the field. In particular, parallel infections using matched parasite lines and culture conditions, including media with and without methylcellulose, could provide a useful reference dataset for the community.

At the same time, such a study would require substantial coordination, reciprocal strain exchange, protocol harmonization and new infection series in two laboratories. It therefore goes beyond the scope of the present Research Advance, which was designed to address the specific methodological concerns raised in response to our original publication. We have restricted our conclusions accordingly and view the proposed collaborative benchmark study as an important direction for future work.
